# Reprogramming resistant genes: in-depth comparison of gene expressions among iPS, ES, and somatic cells

**DOI:** 10.3389/fphys.2013.00007

**Published:** 2013-01-30

**Authors:** Natalia Polouliakh

**Affiliations:** Sony Computer Science Laboratories Inc., Fundamental Research LaboratoriesTokyo, Japan

**Keywords:** iPSC reprogramming, induced/inherited genes, epigenetics, virus type

## Abstract

Transcription factor-based reprogramming reverts adult cells to an embryonic state, yielding potential for generating different tissue types. However, recent reports indicated the substantial differences in pattern of gene expression between induced pluripotent stem (iPS) cells and embryonic stem cells (ESC). In this study, we compare gene expression signatures of different iPS and ES cell lines and relate expression profiles of differently expressed genes to their expression status in somatic cells. As a result, we discovered that genes resistant to reprogramming comprise two major clusters, which are reprogramming dependent “Induced Genes” and somatic origin “Inherited Genes,” both exhibiting preferences in methylation marks. Closer look into the Induced Genes by means of the transcription regulation analysis predicted several groups of genes with various roles in reprogramming and transcription factor DNA binding model. We believe that our results are a helpful source for biologists for further improvement of iPS cell technology.

## Introduction

The ultimate aim of research on induced pluripotent stem cells (iPSC) is to create iPSC that is identical to embryonic stem cells (ESC) and differentiates into tissue specific cell types with intact function. However, recognized discrepancies in gene expression between iPSC and ESC have been reported (Eckhardt, 2006; Chin et al., 2009, 2010; Goldman et al., 2011). Difference in gene expression may reflect difference in methylation, chromatin status, and dynamics of intra-cellular molecular networks and they may affect stem cell behavior in terms of tumorigenicity and spontaneous re-differentiation. Thus, determining the nature of those genes and molecular similarity between different types of pluripotent stem cells is tremendously important.

Chimeric mice generated from iPSC show several abnormalities that are also observed in cloned mice generated by somatic cell nuclear transfer (SCNT), such as high embryonic lethality and shorter life span (Wakayama et al., 1998; Inoue et al., 2002; Ogura et al., 2002; Aoi et al., 2008; Gurdon and Melton, 2008). In global transcriptional profiling of cardiomyocytes induced from iPS and ES cells highly similar expression profiles have been obtained (Gupta et al., 2010). However, a group of fibroblast-associated genes identified overexpressed in iPSC-derived cardiomyocyte beating cluster as compared to their ESC-derived counterparts (Gupta et al., 2010). Another recent study reported that hemangioblastic cells and retinal-pigmented epithelial cells (RPE) derived from human iPS cells exhibit limited expansion in culture and early apoptosis (Feng et al., 2009).

While we are yet to correlate statistics-based computational prediction with molecular features, functional analysis of the iPSC from the variety of somatic sources and reprogramming conditions (Gupta et al., 2010; Polo et al., 2010) remains the foremost way for the verification of the pluripotential stem cell character of each iPSC line. Understanding nature and possible cause and effect of these differences is critically important for developing techniques for derivation of iPSC that are truly identical to ESC.

Given the existence of the reprogramming resistant genes (RRGs) it is important to understand their characteristics, so that a method to overcome somatic cell reprogramming resistance may be developed.

We assume that genes that are differentially expressed between iPS and ES cells are mainly of two categories: the category of iPSC reprogramming process—dependent genes, so called “Induced Genes,” and the category of genes retained from somatic cells due to epigenetic memory, termed “Inherited Gene.” There might be a third category of genes encountering divergences between iPS and ES cells independent on reprogramming, and we do not discuss those genes here, as it requires additional experimental investigations. Regulatory status of Induced Genes can be affected by reprogramming transcription factors, virus vector type, culture conditions, and other factors. Inherited Genes can be considered as a part of transcriptional and epigenetic memory.

Induced Genes category most likely appear through binding of ectopically expressed transcription factors (OCT4, SOX2, KLF4, NANOG, c-Myc) to promoters of their target genes and they can be identified by the computational prediction of transcription factor binding probabilities in promoter regions of target genes.

We used bioinformatics tools to carry out a comparative study of global transcriptional profiles of 13 iPS and 8 ES cell lines. We classified RRGs into Induced and Inherited Genes categories and investigated their role in reprogramming by means of transcription regulation analysis, annotation by H3 histone methylation status in ES cell, promoters CpG density, and correlation with virus type.

## Materials and methods

### Transcriptome data processing

In this study we compared transcriptional profiles from 13 iPS cell lines from 8 published reports hiPS1_8 (Masaki et al., 2008) hiPS2_4 (Masaki et al., 2008), hiPS3_2 (Masaki et al., 2008), BJiPS12 (Maherali et al., 2008), p-iPS01 (Kim et al., 2009), rv-iPS01 (Kim et al., 2009), iPS-PDB-1lox (Soldner et al., 2009), iPS-PDB-2lox (Soldner et al., 2009), Hips7 (Lowry et al., 2008), hiPSC2 (Lowry et al., 2008), hiPS20B1 (Takahashi et al., 2007), hAFF-(4PU)-iPS9 (Zhao et al., 2008), KiPS4F2 (Aasen et al., 2008). Each iPS cell line was compared with the ES cell lines from the same experiment and platform in GEO database (http://www.ncbi.nlm.nih.gov/geo/). Accession number for each DNA chip and induction conditions are summarized in Supplementary Table 5. Raw data files were normalized on GeneSpring (Agilent). First, signal intensities less than 0.01 were set to 0.01, and then each chip was normalized to the 50 percentile of the measurements taken from that chip.

The fold change was calculated relative to the expression level of each gene in the ES cell line, and genes, which are more than 2.0 fold up- or down- regulated in human iPS cells were selected for further analysis. Selected genes were then annotated by their presence (“P”) or absence (“A”) on the respective DNA chip in somatic cells and only genes with consistent status over all cell lines were used for the further analysis.

### Transcription regulation analysis

Regulatory analysis was performed on Induced Genes category with motif discovery tool MEME (Bailey et al., 2006) and ExPlain3.0 suit (Wingender et al., 1996; Kel et al., 2006). Sequences to 5000 base pairs upstream and 500 base pairs downstream from the transcriptional start site (TSS) of the integrated TRANSPro database were used (Wingender et al., 1996).

Human position-specific scoring matrices (PSSM) for OCT4, SOX2, and NANOG were constructed from the regulatory regions of experimentally verified promoters using MEME motif discovery tool (Polouliakh et al., 2005; Bailey et al., 2006). We took this step because human PSSM for those transcription factors do not exist in any database. Following genes were used for the matrices construction: OCT4 matrix from NANOG, OSR2, MSC, KCNN2, PCTK2, RORB; SOX2 matrix from GREG2, SCN3A, NELL1, THBS2, HIST1H4D, INHBA), and NANOG matrix from ONECUT1, GSC, PRKCDBP, FOXB1, FGFR2 (Boyer et al., 2005). Those genes were experimentally verified to allocate respective transcription factor binding sites (TFBS) in their promoters (Boyer et al., 2005). Motif consensus derived from generated matrices are: OCT4 (“TTTGCATT”), SOX2 “(A/G)ACAA(A/T)G”, and NANOG “TAATTG”. KLF4 matrix was acquired from JASPAR database (http://jaspar.cgb.ki.se) and for c-Myc human/mouse TRANSFAC matrix was used.

Matrices of five transcription factors were combined then in one “Master_gene” profile to be used in F-match software within ExPlain3.0 suit (Wingender et al., 1996). F-match evaluates the set of promoters and for each matrix tries to find two thresholds: one, *th*-max, which provides maximum ratio between the frequency of matches in the promoter of in focus (control set, “C”) and background promoters (background, “B”) (over-represented sites); and the second threshold, *th*-min, that minimizes the same ratio (under-represented sites). A binominal distribution of the sites between each control promoter dataset and respective background dataset is calculated and the *p*-value is assigned. For each cell line promoter dataset of up- and down- regulated genes three background datasets of the same size were created using the ExPlain3.0 housekeeping genes set (997 genes) through random selection procedure. Over-represented consensus motifs appeared in three housekeeping background datasets was used as background motifs to calculate *p*-value for the respective motifs in control set using F-match (Wingender et al., 1996).

The selection criteria for the promoter to be accepted, as “*possessing predicted binding motif*” was that the promoter should have at least two out of three main transcription factor binding motifs OCT4, SOX2, NANOG under *p*-value less than 0.001.

### Annotations “*in silico*”

Epigenetic features, such as CpG density and the histone H3 modification status in human ES cells were assigned based on publications (Pan et al., 2007; Weber et al., 2007; Meissner et al., 2008). GO annotations were performed on the manually curated Gene Ontology database within ExPlain3.0 (Wingender et al., 1996). Each cell line was analyzed independently, thus results summarized can have multiple inclusions of the same gene in several groups of Induced Genes category. Pathway search was done on KEGG database using DAVID functional annotation tool (Huang et al., 2009). *T*-test and ANOVA statistical analysis has been performed for calculating virus-type correlation with epigenetic features in down-regulated Inherited Genes category.

## Results

### Global expression comparison of iPS and ES cell lines

**Figure 1:**
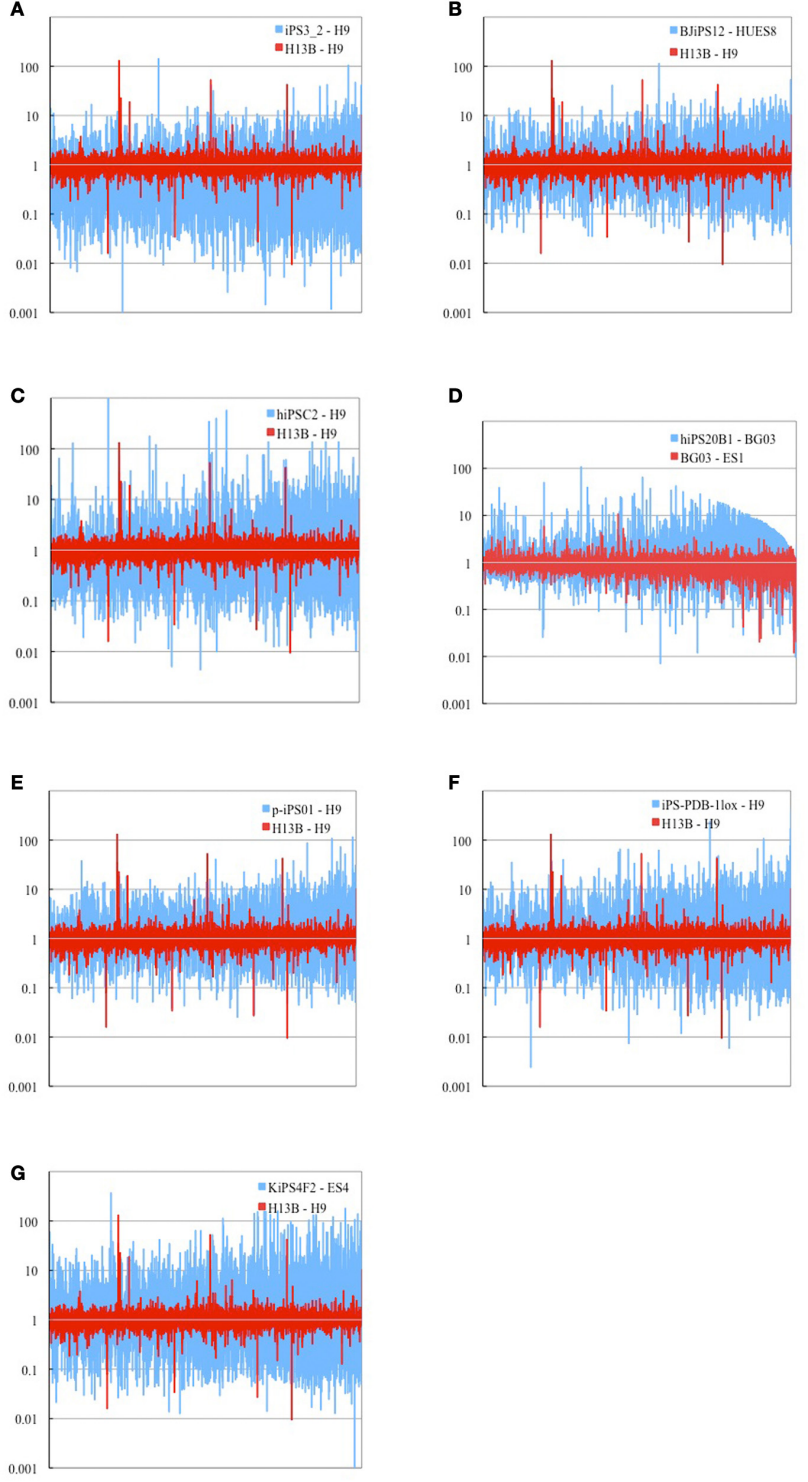
**Logarithm of fold change (FC) comparison of gene expression profiles between representative iPS cell from each of 7 experiments and respective ES cell (blue) and two ES cell lines H13B and H9 obtained from GEO platform 570 (red).** Eight experiments had less number of shared genes (6357) (not shown). In total 9961 genes (15,758 probes) shared among 7 cell lines are shown. **(A)** retorvirus-induced iPS3_2 cell line (Masaki et al., 2008), **(B)** lentivirus-induced BJiPS12 cell line (Maherali et al., 2008), **(C)** retrvorvirus MPx vector-induced hiPS02 cell line (Lowry et al., 2008), **(D)** retrovirus-induced hiPS20B1 cell line (Takahashi et al., 2007), **(E)** virus-free p-iPS01 cell line (Kim et al., 2009), **(F)** cre-recombinase excisable virus-induced iPS-PDB-lox1 cell line (Soldner et al., 2009), **(G)** Mouse stem cell virus-induced KiPS4F2 cell line (Aasen et al., 2008). Genes on each sub-figure are shown in the descending order of expression intensities in human ES cell line BG03. List of cell lines used is represented in Supplementary Table 5.

Comparison of expression profiles has been done on 9961 genes shared by 12 cell lines from 7 experiments (Figures 1A–G) and on 6357 genes for cell line KiPS4F2 (Aasen et al., 2008), as it has less genes shared with other cell lines (Materials and Methods). Genes found over-expressed more than two-folds (FC ≥ 2.0) between iPS and ES cells in more than two cell lines were subjected for categorization into Induced Genes and Inherited Genes according to their status (absence/presence) in the somatic cell of origin.

### Induced genes fall in four groups by transcription analysis

In order to investigate the binding likelihood of transcription factors OCT4, SOX2, NANOG, KLF4, and c-Myc to the promoters of genes in Induced category transcription regulation analysis were performed. For up-regulated Induced Genes category we had 278 genes shared in more than two cell lines and for down-regulated Induced Genes category we had 128 genes shared by more than two cell lines.

Transcription regulation analysis revealed existence of four groups: genes possessing predicted transcription factor binding motifs without significant hit to the particular GO category over a group. We call this group as “master” gene group, and we have in it 42 up-regulated and 25 down-regulated genes shared by more than two cell lines. Second group was genes with significant hit (*p*-value ≤ 0.05) to “development” GO term and predicted transcription factor binding motif (see “Transcription Regulation Analysis” in Materials and Methods). We call this group as “master_development” gene group and have 36 up-regulated genes and 8 down-regulated genes shared by more than two cell lines. Third group was genes with significant hit to GO “development” category and without predicted transcription factor binding motif upon our selection criteria. We call it “development” gene group and have 89 up-regulated genes and 44 down-regulated genes found in more than two cell lines in it. Fourth group was those genes without significant hit to any particular GO term over a group and without predicted transcription factor binding motifs. We call this group “others” and obtained 111 up-regulated genes and 51 down-regulated genes for this group. List of Induced Genes including “master,” “master-development,” “development,” and “others” gene groups is shown in Supplementary Tables 1 and 2 (a–d).

#### “Master” and “master_development” genes are found in each cell line

**Figure 2:**
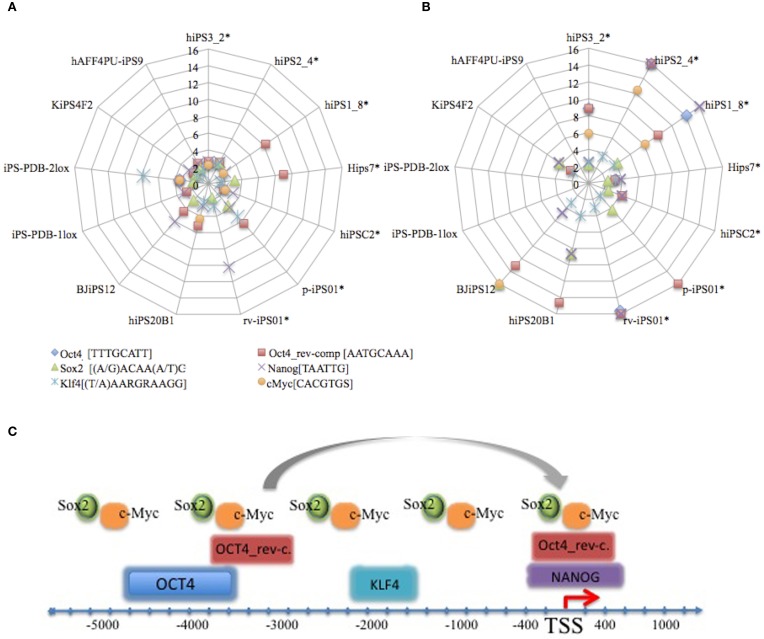
**Distribution of predicted binding motifs over-representation score in the promoters of up- (A) and down-(B) regulated genes comprising “master” and “master_development” groups in 13 cell lines used in our study.** Asterisks (^*^) reference neonate cell lines. Symbols on the graphs correspond to the values represent over-representation score of the motif in the respective cell line (control) comparing to the random set of housekeeping genes (background) (Materials and Methods). Motifs consensus is shown aside the motif name. Number of genes for each cell line can be referenced from Supplementary Tables 1 and 2. IUPAC symbol R and S connoting A/G and C/G, respectively. Panel **(C)** represents promoter regulation model based on the allocations of predicted transcription factor binding sites by F-match tool in ExPlain3.0 suite. TSS denotes transcriptional start site.

We were interested to know the proportion of genes with predicted transcription factor binding motifs in each cell line (“master” and “master development” genes). We noticed that 18% (SD ± 11.01) of up-regulated genes and 23% (SD ± 9.2) of down-regulated genes in each cell line of Induced Genes category belong to “master” and “master_development” groups, i.e., the proportion of such genes in each cell line is similar in size. Then we checked how those transcription factor binding motifs are distributed in each cell line gene group comparing to the random background gene group of the same size (“Transcription Regulation Analysis” in Materials and Methods). Figure 2 represents transcription factors with motif overrepresentation score comparing to the background set in each cell. It is interesting to note that binding affinities of transcription factors in down-regulated groups is significantly higher comparing to up-regulated groups, as it is shown below in Figures 2A,B. Larger fraction of genes with predicted binding motifs in down-regulated “master” and “master-development” groups together with observed stronger binding affinity of those motifs lead us to the conclusion that down-regulation by hypermethylation of CpG islands via the reprogramming must be a major problem to be overcome (Ohi et al., 2011).

NANOG was predicted in the promoters of up-regulated groups of 13 cell lines and in down-regulated groups of 7 cell lines. The fact that NANOG was not one of the reprogramming factors implies the possibility of its ectopic activation in the course of reprogramming, which was also confirmed in other studies (Jiang et al., 2011). Figure 2C depicts promoter model with the approximated transcription factor binding allocation identified in our study. While OCT4 [−4800 (SD ± 200) to −3600 (SD ± 200)], OCT4_rev-comp.pos1 [−3700 (SD ± 1000) to −2700 (SD ± 800)], OCT4_rev-comp.pos2 [−400 (SD ± 100) to + 500 (SD ± 0.0)], KLF4 [−2000 (SD ± 600) to −1600 (SD ± 700)], and NANOG [−400 (SD ± 100) to +400 (SD ± 100)] allocations to the TSS are comparatively constant, SOX2, and c-Myc positioning was not related to TSS. Reverse-complementary predicted sites of OCT4 were found in all cell lines.

#### Epigenetics of induced gene category

To examine differences among groups and categories regarding the histone H3 epigenetic modifications and CpG promoter density of genes included (Pan et al., 2007; Weber et al., 2007; Meissner et al., 2008) we performed chi-square independence test (Yates correction) to identify correlations based on these epigenetic features.

Chi-square test indicated prevalence of genes with bivalent (H3K4K27, trimethylation of both lysines K4 and K27) modification status of promoters of the Induced Gene category (*p*-value ≤ 0.002) as compared to the promoters of the Inherited Gene category when compared between two categories.

In Induced Genes category, genes with bivalent modification (H3K4K27), unmethylated genes and genes with non-defined methylation “ND’ status in total constitute same proportion of 60% (SD ± 0.1) in up-regulated genes in each cell line and similar proportion of 71% (SD ± 7.1) of down-regulated genes in each cell line, independent on group. Gene lists with respective annotations are shown in Supplementary Tables 1 and 2 (a–d).

Comparing groups within Induced Genes category we identified that as up-regulated “development” group as down-regulated “development” group are enriched in High CpG promoters (HCP) when compared to “master” and “master_development” groups with *p*-values less than 0.001 and less than 0.01, correspondingly. It has been shown in previous study (Meissner et al., 2008) that *in vitro* culture induces hypermethylation of housekeeping genes coded by HCP promoter. These genes are associated with ubiquitous housekeeping genes and key developmental genes (Saxonov et al., 2006); both are highly expressed in ESCs.

“Master” and “master_development” groups of Induced Genes category with predicted binding sites for reprogramming transcription factors have higher inclusion of Low CpG promoters (LCP), Intermediate CpG promoter (ICP) and promoters with non-defined CpG density (ND), in comparison with “development” and “other” groups under *p*-value of less than 0.05, where transcription factor binding motifs were not predicted.

We conclude that transcriptional activity status of CpG-poor promoters hypermethylated in somatic cells is not precluded on the first stage of the transfection, and consequently these genes are showing more plastic response to re-programming in comparison with High CpG less methylated and inactive promoters (Pan et al., 2007).

#### Pathways in induced genes category

Following pathways (Huang et al., 2009) were detected in up- and down-regulated or either group in Induced Genes category: calcium signaling pathway (4.00E-03, up-, down-), cell adhesion molecules CAMs pathway (1.27E-02, up-), PPAR signaling pathway (1.83E-02, up-), Tight junction (2.69E-02, down-), neuroactive ligand-receptor interaction (3.45E-02, down-) pathways. Calcium related genes in both up- and down-regulated groups might imply the possibility for induction of differentiation and development.

### Inherited genes of somatic transcriptional memory

Inherited Genes are those expressed in somatic cell and up- or down-regulated in iPSC comparing to ES. They are mainly of two origins: (a) those retaining their methylation status from somatic substrates and (b) those activated or repressed through the viral transduction in iPSC in the course of reprogramming. We identified and categorized 1367 up-regulated genes and 1113 down-regulated genes of Inherited Genes category focusing on genes found in more than 3 cell lines (Supplementary Tables 3 and 4). Inherited Genes includes half of genes with univalent (H3K4, trimethylation of lysine residue 4) modification status in ES cell genes with *p*-value less than 0.001 against Induced Genes category by chi-square independence test. Recent observation showed that reprogramming a somatic cell into a pluripotent state generates hundreds of aberrantly methylated loci, predominantly at CpG islands, and associated with genes (Meissner et al., 2008), thus this category of Inherited Genes including many de-methylated loci is the most prone to reprogramming (Polo et al., 2010).

iPSC up-regulated group of Inherited Genes category (Supplementary Table 3) includes IGF2 parentally expressed and H19 maternally expressed imprinted genes, shared by 3 and 5 different iPSC lines, respectively, and several IGF-binding proteins: IGFBP7 (7 cells), IGFBP3 (6 cells), IGFBP5 (5 cells), IGFPB6 (3 cells). In the embryo, imprinted genes regulate growth and development. Postnatally, imprinted genes control behavior, which may also affect the flow of nutrients from the mother to the developing pup (Davis and Uthus, 2003; Haig, 2004). Increased activity of the IGF2 gene has been associated with many types of cancer (Chao and D'Amore, 2008).

#### Pathways in inherited genes category

Following pathways (Huang et al., 2009) were detected in up- and down-regulated or either group in Inherited Genes category: Focal adhesion (4.28E-03, up-, down-), ECM-receptor interaction (7.71E-07, up-), p53 signaling pathway (1.97E-02, up-, down-), Pathways in cancer (2.83E-05, up-, down-), Melanoma (9.36E-04, down-), Lysosome (3.39E-03, up-), Apoptosis (5.47E-03, up-, down-), Prostate cancer (1.70E-02, down-), Bladder cancer (6.65E-03, up-), Small cell lung cancer (2.82E-02, down-), Colorectal cancer (4.09E-03, up-), and Tight junction (7.13E-03, up-, down-). Genes from these groups may affect carcinogenic nature of iPS cells.

#### Virus type correlation in down-regulated group of inherited genes

**Figure 3:**
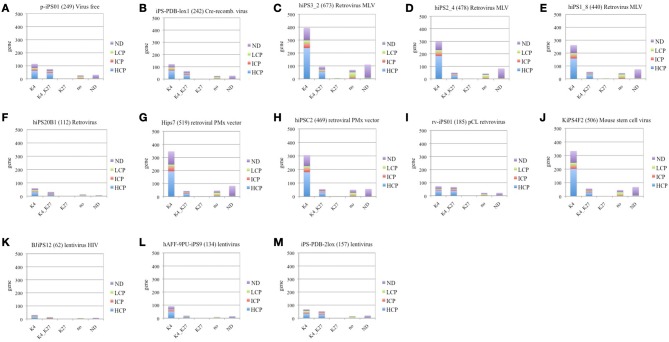
**Classification of iPS cell down-regulated Inherited genes according to the histone H3 methylation status in ES cell and CpG promoter density. (A)** virus free **(B)** cre-recombinase excisable virus, **(C–J)** retrovirus, and **(K–M)** lentivirus. Figure legends specifications for methylation status of histone 3 (H3) in ES cell: “K4”—trimethylation of lysine on residue 4, “K4_K27”—trimethylation of lysine on residue 4 and residue 27, “K27”—trimethylation of lysine on residue 27, “no”-no methylation marks onH3, “ND”—undefined methylation status. Figure legends specifications for the promoter CpG density in ES cell: “NO”, undefined CpG promoter density; LCP, Low CpG promoter density; ICP, intermediate CpG promoter density; HCP, high CpG promoter density.

Here we investigate correlations between virus-type and number of down regulated genes of Inherited category in each cell line. Figure 3 represents down-regulated Inherited category genes shared by more than 3 cell lines and annotated by promoter CpG density and histone H3 modification status in ES cells. List of Inherited down-regulated genes is represented in Supplementary Table 4 (a–c).

We have following cell lines: 2 virus-free cell lines, such as p-iPS01 (249 genes), iPS-PDB-lox1 (242 genes); 8 retrovirus cell lines, such as, hiPS3_2 (673 genes), hiPS2_4 (478genes), hiPS1_8 (440 genes), hiPS20B1 (112 genes), Hips7 (519 genes), hiPSC2 (469 genes), rv-iPS01 (185 genes), KiPS4F2 (506 genes), and 3 lentivirus cell lines, such as BJiPS12 (62 genes), hAFF-4PU-iPS9 (134 genes), and iPS-PDB-2lox (157 genes).

The lowest number of down-regulated RRGs was found in following iPSC lines: BJiPS12 (62 genes), hiPS20B1 (112 genes), hAFF-4PU-iPS9 (134 genes), and iPS-PDB-2lox (157 genes), where last three cell lines are derived through the lentiviral transduction. Cre-recombinase excisable virus induced cell line iPS-PDB-lox1 (242 genes) and virus-free cell p-iPS01 (249 genes) had comparatively low number of down-regulated genes, but still higher than lentivirus induced cells BJiPS12 (62 genes), hAFF-4PU-iPS9 (134 genes), and iPS-PDB-2lox (157 genes) (Figure 3). While the transcription factors used for reprogramming can be excised by inducible gene expression once reprogramming is established (Pan et al., 2007; Jiang et al., 2011), residual sequences and chromosomal disruptions may still result in harmful alterations that could pose clinical risks. Retrovirus induced cell line rv-iPS01 (185 genes) produced in the same experiment (Kim et al., 2009) with straightforward protein delivery inducted cell line p-iPS01 (249 genes) show slightly lower fraction of down-regulated genes, while the opposite tendency is expected.

The rest of cells produced by retroviral induction show approximately same level of RRGs (510 genes, SD ± 82). It is known that the MLV retrovirus vector integrates near transcription start sites and CpG islands, while the HIV lentivirus vector integrates preferentially within active transcription units (Lewinski et al., 2006; Woltjen et al., 2009). Based on the results we assume that integration of retrovirus in the proximity of TSS of the dividing cells can significantly increase the probability of stochastic silencing, which results in larger fraction of genes down-regulated.

*T*-test showed statistical difference on genes with H3K4 methylation status with *p*-value less than 0.02, when retrovirus group and virus-free groups were compared. Therefore, we assume the higher plasticity of virus-free derived cells, as they are having less number of housekeeping and key developmental genes with H3K4 methylation status in ES cell.

ANOVA analysis (*p*-value ≤ 0.05) for promoter density characteristics over virus-free, retrovirus, and lentivirus groups showed difference in distribution for ICP genes and genes with undefined CpG density (“ND”), which is also reflected in recent publications (Pan et al., 2007; Weber et al., 2007; Meissner et al., 2008).

Most of down-regulated groups enriched with genes having the univalent (H3K4) modification status in human ES-cells and HCP (Figures 3E,J,H,G) are those observed in iPSCs generated by retroviral or lentiviral transduction. Virus-free cell lines have higher fraction of genes with bivalent (H3K4K27) modification status in human ES cells, i.e., those, possibly exhibiting higher plasticity to reprogramming. These finding can help biologists to select the appropriate virus for the analysis.

Three cell lines of neonatal somatic cell origin, produced in the same experiment (Masaki et al., 2008) with different passage of length hiPS3_2 80 days (673 genes), hiPS2_4 102 days (478 genes), and hiPS1_8 180 days (440 genes) show gradual decrease in down-regulated gene number with the increase of passage number, which correlated with previous findings (Chin et al., 2010; Ohi et al., 2011).

## Discussion

Genes showing statistical difference in expression between iPSCs and ESCs and shared by several iPSC lines can be considered as RRGs. RRGs were classified in two categories of Induced Genes and Inherited Genes depending on their expression status in somatic cells of origin. Induced Genes exhibit bivalent (H3K4K27) modification status in ES cells with the predominance of Intermediate and Low CpG density promoters and promoters with non-defined CpG density (“ND”).

Faulty resetting of inactive yet “posed” state of bivalent domains is critical for the consequent differentiation of iPS cell in tissue (Kim et al., 2010). On the contrary, Inherited Genes category was enriched in univalent (H3K4) modification status in ES cell and showed preponderance for High CpG density promoter genes.

Part of our Inherited Genes category was identified as fibroblast-associated in prior studies (123 genes), while Induced Genes category associated with iPS-specific reprogramming network showed tiny overlap (8 genes) of our genes with resistant genes known from other studies (Chin et al., 2010; Gupta et al., 2010; Newman and Cooper, 2010; Lister et al., 2011; Ohi et al., 2011). The analysis of iPSC-differentiated cardiomyocytes beating clusters reported by Gupta et al. showed significant overlaps with our results in 111 genes (87 up-, 24 down-) with our Inherited Genes category and only 6 genes in Induced category (Supplementary Tables 1, 2, 3, 4 “overlap” columns). Ten genes (COMP1, DYNLT3, NME4, OXCT1, MGMT, PTGR1, MGC3207, CKLF, ZNF167, ZNF626) from our study were verified in functional analysis by qPCR to be over-expressed in somatic cell and continue their *up*-regulation in iPSC-derived cardiomyocyte beating clusters (Gupta et al., 2010). Seven out of 15 genes reported as differently expressed between iPS and ES cell lines over four laboratories in the study of Newman and Cooper also found in Inherited (6 genes) and Induced (1 gene) Gene categories in our study.

CSRP1 (cystein and glycine rich protein 1, 6 cell lines, neonate, and adult), COMT (catecol-O-methyltransferase, 5 cell lines, neonate and adult) in Inherited up-regulated group (Supplementary Table 3a) and C9orf64 (5 cell lines, neonate) Inherited down-regulated group (Supplementary Table 4a) are top somatic cell genes expressed in iPS cell (Ohi et al., 2011) also identified in our study. CAT (catalase) (Warren et al., 2010) fibroblast-associated gene is top shared gene (9/13 cell lines) in our Inherited up-regulated gene group (Supplementary Table 3a). Results of another functional analysis (Lister et al., 2011) overlapped with 5 genes of our list of Inherited Genes. This finding let us conclude about the higher plasticity of Induced Genes to reprogramming and consequent differentiation into somatic cell in comparison with Inherited somatic memory genes.

Transcriptional analysis of Induced Genes category revealed similar fraction of genes with predicted transcription factor binding motifs in up- and down-regulated groups in each cell line. Stronger binding affinity was observed in down-regulated groups of all cells suggesting that stochastic genes silencing should be the major problem to overcome for more successful reprogramming. Predicted NANOG binding motif in the proximity (−500 to +500) of TSS in the most cell lines leads us to the conclusion about its ectopic activation. OCT4 and KLF4 are comparatively regular in binding allocation, while SOX2 and c-Myc did not show any consistency in our study. All predicted TFBS are distantly allocated, which is consistent with the recent experimental publication (Soufi et al., 2012).

Concerning the general characteristics of RRGs on the pathway level, Inherited Gene category included cancer and apoptosis-related pathways, such as focal adhesion, p53 signaling pathway, which also observed in the recent results (Soufi et al., 2012). This may affect unwanted tumorigenic propensity of iPS cells and further experimental verification of this issue is required. Induced Gene category was enriched in calcium-signaling pathway, cell adhesion, PPAR signaling, and tight junction. These pathways may contribute to the embryogenesis, development, and immune response, but biological implication of such differential expressions is yet to be elucidated. It is evident that substantial numbers of genes are differentially expressed due to various factors leading to differences between iPS and ES cells.

Pertaining to the virus type two conclusions can be drawn. First, virus-free and lentivirus-derived iPS cell lines have lower number of down-regulated Inherited Genes, while retrovirus insertion in the promoters of genes seems to provoke strong inhibitory effect. Remarkably, virus-free and lentivirus-derived iPS cell lines have larger number of genes with bivalent (H3K4K27) modification status in ES cells, i.e., under these conditions somatic cells exhibit higher susceptibility to reprogramming than those generated through the retrovirus transduction. Second conclusion is that passage length anti-correlates with number of RRGs.

For further improvement of iPSC technology several factors should be taken into the consideration. Basing on the status of gene in somatic cell of origin two natures of RRGs should be considered: Induced Genes active in somatic substrate and Inherited Genes inactive in somatic substrate. Tiny fraction of Induced Genes (bivalent H3K4K27, LCP/ICP) was identified up-/down-regulated in iPS-derived somatic cell (Gupta et al., 2010). This means that they might be activated during the first stage of reprogramming (Soufi et al., 2012), which make us suggest that longer passage can help to resolve this problem. More attention should be paid to the Inherited Genes category (univalent H3K4, HCP), retaining somatic cell transcriptional memory. They were abundantly found in iPS-derived somatic cell (Gupta et al., 2010) and overlap with RRGs from other studies (Chin et al., 2010; Newman and Cooper, 2010; Lister et al., 2011; Ohi et al., 2011). Active and demethylated High CpG density promoters might attract retrovirus insertion, which causes consequent silencing of the promoter through *de novo* methylation. Virus-free or miRNA-mediated (Ankye-Danso et al., 2011) reprogramming may be more plausible in the future. For the future work it is important to identify key transcription factors within Inherited Genes category to be able to reduce/block their activity. Donor age and developmental stage is important for the selection of somatic cell substrate. While heterogeneous tissue culture does not simply reflect the epigenetics status of the substrate cell, several reports indicate that somatic cell/progenitor cells can be epigenetically favored substrates for nuclear resetting (Aasen et al., 2008; Silva et al., 2008).

The influence of RRGs on the intactness of function after the consequent differentiation iPSCs in organs and tissues is extremely important for the validation and standardization of iPSC technology, and our results can be a help for this.

### Conflict of interest statement

The author declares that the research was conducted in the absence of any commercial or financial relationships that could be construed as a potential conflict of interest.
